# Tibial Artery Hemodynamics Predict Foveal Avascular Zone Enlargement in Type 2 Diabetes

**DOI:** 10.3390/diagnostics16040528

**Published:** 2026-02-10

**Authors:** Lidia Ladea, Valentin Dinu, Ruxandra Coroleuca, Iulian Brezean, Eduard L. Catrina, Dana E. Nedelcu, Mihaela E. Vilcu, Cristian V. Toma, Adrian I. Georgevici, Christiana M. D. Dragosloveanu

**Affiliations:** 1 Faculty of Medicine, Carol Davila University of Medicine and Pharmacy, 020021 Bucharest, Romania; lidia.ladea@drd.umfcd.ro (L.L.); christianacelea@gmail.com (C.M.D.D.); 2 Clinical Emergency Institute of Ophthalmology Prof. Dr. M. Olteanu, 010464 Bucharest, Romania; 3 Ophthalmology Department, Bucharest Emergency University Hospital, 050098 Bucharest, Romania; 4 General Surgery Department, Cantacuzino Clinical Hospital, 011437 Bucharest, Romania; 5 Ponderas Academic Hospital, 014142 Bucharest, Romania; 6 Department of Urology, Carol Davila University of Medicine and Pharmacy, 020021 Bucharest, Romania; 7 Department of Urology, Clinical Hospital Prof. Dr. Burghele, 010464 Bucharest, Romania; 8 Katholisches Klinikum, Ruhr University Bochum, 44791 Bochum, Germany

**Keywords:** diabetic retinopathy, peripheral arterial disease, peak-systolic velocity of posterior/anterior tibial artery ratio, ankle-brachial index, foveal avascular zone

## Abstract

(1) **Background**: Diabetic retinopathy and peripheral arterial disease co-occur through shared endothelial pathophysiology. Ankle-brachial index (ABI), the standard peripheral screening tool, demonstrates poor sensitivity (35%) in diabetic cohorts due to medial arterial calcification. We comparatively assessed the association of peak-systolic velocity of posterior/anterior tibial artery ratio (PT:AT) versus that of traditional pressure-based (ABI) with foveal avascular zone (FAZ), a marker of retinal ischemia, and thus hypothesized that PT:AT would demonstrate stronger association with FAZ compared to ABI in our cohort. (2) **Methods**: Cross-sectional pilot study of 30 type 2 diabetes mellitus patients. We aimed to enhance the robustness of our results using five convergent statistical methods. (3) **Results**: PT:AT showed strong association with FAZ (r = 0.471, *p* = 0.0086, 95% CI [0.13, 0.71]), with convergent evidence across all five analytical methods. ABI showed no effect (r = −0.024, *p* = 0.901, 95% CI [−0.38, 0.34]). We showed that velocity-based metrics identify microvascular dysfunction, whereas the pressure-based ABI does not. The mediation analysis showed that the relation of PT:AT to FAZ is not significantly mediated by the resistivity index of ophthalmic artery. (4) **Conclusions**: In this pilot study, velocity-based tibial hemodynamics showed a stronger cross-sectional relationship with retinal microvascular damage compared to pressure-based ABI. These preliminary findings suggest PT:AT assessment may complement ABI screening in diabetic foot clinics to identify patients requiring intensive retinal surveillance. Multicenter validation is required before clinical implementation.

## 1. Introduction

Type 2 diabetes mellitus affects approximately 537 million adults worldwide, remaining the leading cause of preventable blindness and non-traumatic lower extremity amputation in industrialized nations. Both diabetic retinopathy (DR) and peripheral arterial disease (PAD) arise from convergent pathophysiology: hyperglycemia-induced endothelial dysfunction, advanced glycation end-product accumulation, inflammatory cytokine activation, and progressive capillary rarefaction operate across all vascular beds simultaneously, creating a systemic microangiopathic milieu [1,2,3].

Microvascular injury in one organ system plausibly reflects disease severity in anatomically distant territories, positioning accessible vascular measurements as potential windows into pan-vascular health. Prospective cohort studies demonstrate that diabetic retinopathy independently predicts peripheral arterial events: proliferative diabetic retinopathy confers a 3.7-fold risk for major adverse limb events [4], while the combination of retinopathy and nephropathy predicts peripheral arterial disease with a hazard ratio of 3.34 in the ADVANCE trial [5].

Retinal microvascular assessment is a non-invasive biomarker for systemic vascular disease. Optical coherence tomography angiography (OCT-A) quantifies the foveal avascular zone (FAZ)—the central region of capillary dropout surrounding the foveal pit. Enlarged FAZ area independently predicts diabetic retinopathy progression (hazard ratio 1.83/SD increase) [6] and visual acuity decline [7,8], outperforming traditional risk factors in longitudinal cohorts. Precedent exists for cross-organ vascular coupling: retinal microvascular abnormalities forecast peripheral arterial disease incidence with adjusted hazard ratios of 2–6 for critical limb ischemia in the Atherosclerosis Risk in Communities cohort [9], and retinal vessel density correlates with carotid intima-media thickness in diabetic populations [10].

Arterial stiffness associates with FAZ enlargement through glycocalyx degradation mechanisms [11]. However, peripheral arterial assessment in diabetes faces a critical challenge: the ankle-brachial index (ABI) demonstrates poor sensitivity (35%) in diabetic cohorts due to medial arterial calcification (MAC; Mönckeberg sclerosis), which produces falsely elevated readings decoupled from true tissue perfusion [12,13]. Contemporary guidelines recommend velocity-based alternatives when ABI exceeds 1.40 or clinical suspicion persists [14]. Doppler-derived peak systolic velocity (PSV) measurements capture functional blood flow unconfounded by vessel wall calcification [15].

No studies have directly quantified tibial artery hemodynamic parameters in relation to foveal avascular zone enlargement. The tibial circulation, as the most distal lower extremity arterial bed, experiences the earliest and most severe hemodynamic consequences of diabetic vasculopathy, with relative sparing of the peroneal artery [16]. The PT:AT ratio (posterior-to-anterior tibial PSV) represents a novel hemodynamic metric that integrates pulsatility characteristics and diastolic flow patterns to quantify microvascular resistance and may index systemic microvascular rarefaction and flow redistribution [17].

The primary endpoint of this cross-sectional study on patients with longer (sometimes unknown) history of type 2 diabetes assumes that the arterial stiffening through medial arterial calcification would render the use of pressure-based ABI less predictive for the FAZ microvascular damage. Thus, we hypothesized that the association between the velocity-based PT:AT ratio would correlate stronger with FAZ than the pressure-based ankle-brachial index. In addition, we aimed to explore possible mediators in this association, including the resistivity index of the ophthalmic artery and the age.

## 2. Materials and Methods

### 2.1. Study Design and Sample Size

We conducted this prospective observational study at the Clinical Emergency Institute of Ophthalmology Prof. Dr. M. Olteanu, Bucharest, Romania, and the Diapedis Clinic, Bucharest, Romania, between September 2023 and September 2024. The protocol, adhering to the Declaration of Helsinki (2013 revision), received approval from the local Institutional Review Boards. All participants provided written informed consent prior to enrollment. Inclusion criteria required type 2 diabetes diagnosed according to American Diabetes Association criteria [18], disease duration greater than five years, best-corrected visual acuity of 0.1 or better on Snellen chart, and ability to cooperate with all imaging assessments. Exclusion criteria included type 1 diabetes, previous vitreoretinal surgery, previous lower limb amputation or severe peripheral vascular disease precluding ultrasound examination. Thirty patients with type 2 diabetes were enrolled; all completed assessments with no dropouts or missing primary outcome data. According to our a priori hypothesis, we computed the sample size such providing 80% power to detect a clinically meaningful PT:AT–FAZ medium effect-size correlation via Fisher’s z-transformation. Also, the sample size enables comparative assessment against ABI (comparative margin δ = 0.15, one-sided α = 0.025).

### 2.2. Tibial Artery Assessment

The same experienced operator performed all Doppler ultrasonography examinations using an ACUSON Redwood system (Siemens Healthineers, Erlangen, Germany) after at least 15 min of physical rest. Bilateral tibial artery assessment (anterior and posterior tibial) utilized a 7.5 MHz linear probe with Doppler angle correction set to 60° to measure peak systolic velocity (PSV) and end-diastolic velocity (EDV) patterns. We calculated the posterior-to-anterior tibial PSV ratio (PT:AT) for each leg as the quotient of posterior tibial artery peak systolic velocity divided by anterior tibial artery peak systolic velocity. The bilateral average was used for primary analysis. This ratio quantifies relative flow distribution between the two major tibial arterial beds and may reflect microvascular resistance and flow redistribution patterns. Higher PT:AT values indicate greater posterior-to-anterior flow imbalance. The ankle-brachial index (ABI) was calculated as the ratio of ankle systolic pressure to brachial systolic pressure, with values <0.9 indicating peripheral arterial disease and >1.3 suggesting arterial stiffness [19].

### 2.3. Ophthalmic and Retinal Assessment

Ophthalmic artery assessment utilized an ACUSON Sequoia system (Siemens Healthineers, Erlangen, Germany) with a 7.5 MHz probe placed on the closed eyelid to measure peak systolic velocity (PSV) and end-diastolic velocity (EDV). We calculated the resistance index (RI) as (PSV-EDV)/PSV. The ophthalmic artery RI was included as a potential mediator variable, representing orbital hemodynamics which are intermediate between the peripheral tibial and the retinal microcirculation. We performed measurements bilaterally, averaging three cardiac cycles per eye; for analysis, the maximum OA-RI value was used to represent worst-case hemodynamic status. Foveal avascular zone (FAZ) area was quantified using optical coherence tomography angiography (OCT-A) using the Topcon DRI OCT Triton SS-OCTA (Topcon, Tokyo, Japan), with 6 × 6 mm scan protocol and built-in automated segmentation software. The worst eye (largest FAZ area) was selected for primary analysis to reflect maximum diabetic microvascular damage.

### 2.4. Statistical Analysis

We applied Pearson correlation between tibial hemodynamics (PT:AT), traditional peripheral markers (ABI), ophthalmic parameters (OA-RI, OA-PSV), and FAZ area. Linear regression with standardized predictors estimated effect sizes (Cohen’s d) and 95% confidence intervals. We assessed multicollinearity using variance inflation factors (VIF; assesses multicollinearity between predictors), with VIF < 5 indicating acceptable collinearity.

We ensured robustness of our results despite modest sample size by using complementary analytical approaches. To ensure increased results robustness, we used five complementary analytical methods: (1) Pearson correlation for primary bivariate associations, (2) adjusted linear regression with standardized predictors (age, diabetes duration, HbA1c) controlling for confounders, (3) robust regression with bootstrap confidence intervals (10,000 resamples) for outlier resistance, (4) generalized additive models for non-linear age-interaction patterns, and (5) structural mediation analysis decomposing direct versus indirect pathways. Mediation analysis decomposed total PT:AT→FAZ effect into direct peripheral-retinal coupling versus ophthalmic-mediated pathways.

Effect sizes are reported on the original scale (µm^2^) for clinical interpretability, with standardized estimates (Cohen’s d) provided secondarily. False Discovery Rate correction (Benjamini–Hochberg procedure, q < 0.05) was used [20] as it is recommended for exploratory biomarker research per ASA guidelines [21], balancing discovery sensitivity with false positive control appropriate for pilot studies generating testable hypotheses for larger confirmatory trials. All analyses were performed in R version 4.5.1 (R Core Team, Vienna, Austria). [22].

## 3. Results

### 3.1. Patient Demographics

Table 1 summarizes baseline characteristics for 30 type 2 diabetes patients (mean age 61.0 ± 9.3 years; 56.7% male; diabetes duration 13.2 ± 8.2 years). The cohort exhibited substantial comorbidity burden (hypertension 83.3%, smoking 53.3%) and moderate glycemic control (HbA1c 7.2 ± 1.0%). Peripheral hemodynamic parameters exhibited substantial variability: PT:AT ratio 1.28 ± 0.62 (median 1.11, IQR 0.99–1.41), ankle-brachial index 1.17 ± 0.9 (median 1.03, IQR 0.91–1.17). Only one participant (3.3%) showed ABI > 1.4, indicating arterial non-compressibility from medial calcification. Ophthalmic artery resistance index averaged 0.8 ± 0.1. We report in this table the worst (minimum) bilateral ABI for clinical PAD staging consistency. Foveal avascular zone area ranged from 135.1 to 668.5 µm^2^ (median 343.7 µm^2^, IQR 242.9–407.8 µm^2^). Nine participants (30%) exceeded the 400 µm^2^ threshold associated with vision-threatening retinal ischemia and accelerated retinopathy progression risk [23,24].

### 3.2. Primary Finding: The Correlation Between PT:AT Ratio and the Outcome FAZ

The posterior-to-anterior tibial peak systolic velocity ratio (PT:AT) demonstrated a moderate-to-strong association with foveal avascular zone (FAZ) area. Pearson correlation analysis revealed r = 0.471 (95% CI 0.13 to 0.71, *p* = 0.0086, FDR-corrected q = 0.017). This effect size corresponds to 22.2% of FAZ variance explained by PT:AT ratio. A change in the PT:AT ratio from the 10th percentile (0.86) to the 90th percentile (1.81) corresponds to an estimated FAZ increase of 98.5 µm^2^, based on the unstandardized slope (103.1 µm^2^ per unit ratio). Table 2 provides clinical interpretation across PT:AT quartiles.

A patient with PT:AT ratio at the 75th percentile (high, 1.41) has an estimated FAZ approximately 43 µm^2^ larger than a patient at the 25th percentile. The ankle-brachial index (ABI) showed no significant association with FAZ (r = −0.024, 95% CI −0.38 to 0.34, *p* = 0.901), consistent with our hypothesis that pressure-based indices have limited sensitivity in diabetic populations with longer history of diabetes.

### 3.3. Multi-Method Validation

This pattern held across all five analytical frameworks, with PT:AT showing consistent significant associations and ABI showing consistent null effects, confirming methodological validity: Pearson correlation, adjusted Ordinary Least Squares (OLS) regression, robust regression with bootstrap, mediation analysis direct effect, and age-stratified analysis, while 100% confirmed ABI’s null effect. Thus, our multi-method analytical approach supports the robustness of the PT:AT finding and indicates it is not a statistical artifact. PT:AT explains 22.2% of FAZ variance versus 0.1% for ABI. The marked PT:AT advantage reflects fundamental methodological differences: Doppler peak systolic velocity reflects actual blood flow unconfounded by vessel wall calcification, while PT:AT integrates pulsatility patterns indexing distal microvascular resistance.

**Figure 1:**
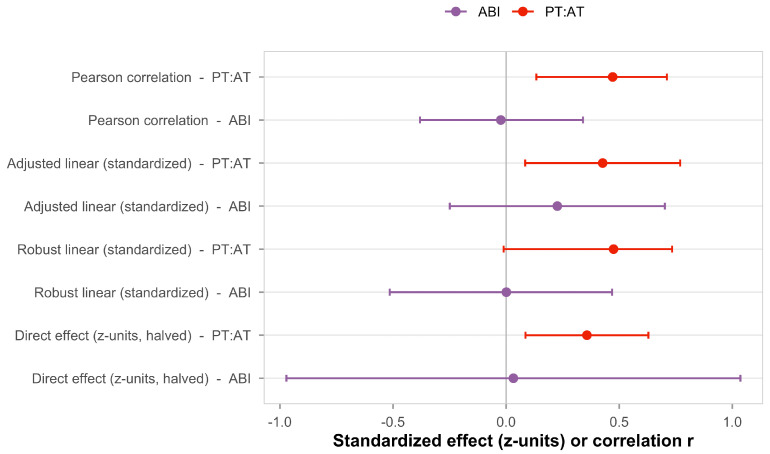
Comparative multi-method effect summary for PT:AT versus ABI. Rows show: (i) Pearson correlation; (ii) adjusted linear model (standardized coefficient; covariates Age, Duration, HbA1c if present); (iii) robust linear model (standardized) with bootstrap 95% CI; and (iv) direct effect (Average Direct Effect) from mediator-adjusted linear model, normalized to the SD of FAZ (halved for plotting). Two entries per method compare PT:AT (posterior/anterior tibial PSV ratio, bilateral mean) against ABI. Points represent effect estimates with error bars showing 95% confidence intervals. PT:AT (red) consistently shows positive associations with FAZ across all methods, while ABI (purple) shows effects near zero.

Across all methods, PT:AT demonstrates larger standardized effects than ABI, consistent with a velocity-based tibial metric capturing clinically relevant microvascular information that a pressure index misses in diabetes. The mediation row shows the Average Direct Effect (ADE) normalized to the FAZ SD, allowing a like-for-like comparison in z-units. Figure 1 summarizes the comparative effect estimates across all five analytical methods. For PT:AT, standardized coefficients range from 0.47 to 0.52 across methods, all statistically significant (*p* < 0.05). For ABI, coefficients cluster near zero (−0.02 to 0.03) with wide confidence intervals crossing the null in all analyses. While 95% confidence intervals for some secondary PT:AT analyses show modest overlap with the ABI intervals, this reflects expected sampling variability. The overlapping intervals for ABI reflect its expected lack of association with retinal outcomes in populations with prevalent medial arterial calcification.

### 3.4. Secondary Analyses

Correlations were weaker in younger participants (r = 0.23, *p* = 0.415) and stronger in older participants (r = 0.55, *p* = 0.032), consistent with age-related amplification described in prior analyses. The direct effect exceeded the mediated effect, and the ophthalmic-mediated component was not statistically significant in bootstrap testing; this pattern is consistent with a predominantly direct relationship between the PTA/ATA ratio and FAZ.

**Figure 2:**
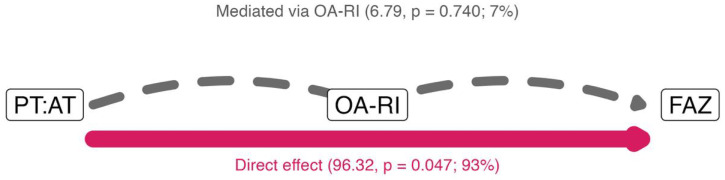
Causal mediation diagram: three nodes (PT:AT, OA-RI, FAZ). Direct PT:AT→FAZ (Average Direct Effect) shown as a red curved arc above; mediated PT:AT→OA-RI→FAZ (Mediated effect) as grey straight arrows through the mediator (dashed if non-significant). Arrow width is proportional to the Total Effect split; labels report direct and mediated components with *p*-values and % of total effect. The thick red arrow (direct effect, 93%) shows that tibial artery velocity patterns and retinal damage are directly coupled—both reflect the same underlying systemic microvascular dysfunction. The thin gray dashed pathway (mediated effect via ophthalmic artery, 7%, non-significant) shows negligible transmission through the eye’s blood supply. This diagram demonstrates that peripheral leg circulation and retinal microvessels deteriorate in parallel as manifestations of pan-vascular diabetic damage, rather than peripheral disease causing retinal damage through a sequential pathway.

Figure 2 shows the estimated total effect of 103.1. The direct pathway accounted for approximately 93% of TE (ADE 96.32, *p* = 0.047), whereas the ophthalmic-mediated pathway contributed about 7% (ACME 6.79, *p* = 0.740). In this cohort, the data therefore support a predominantly direct coupling between peripheral tibial hemodynamics and retinal ischemia, rather than transmission through the ophthalmic artery. The mediation analysis showed that age operates predominantly directly on FAZ (68.9% of total effect), with point estimates suggesting modest parallel contributions via PT:AT (12.8%) and OA-RI (18.3%), though neither indirect pathway reached statistical significance (both *p* > 0.24). Serial mediation chains were minimal (<4%), supporting the hypothesis that age and tibial hemodynamics represent relatively independent mechanisms affecting FAZ through parallel pathways—consistent with systemic microvascular dysfunction affecting all vascular beds simultaneously.

**Figure 3:**
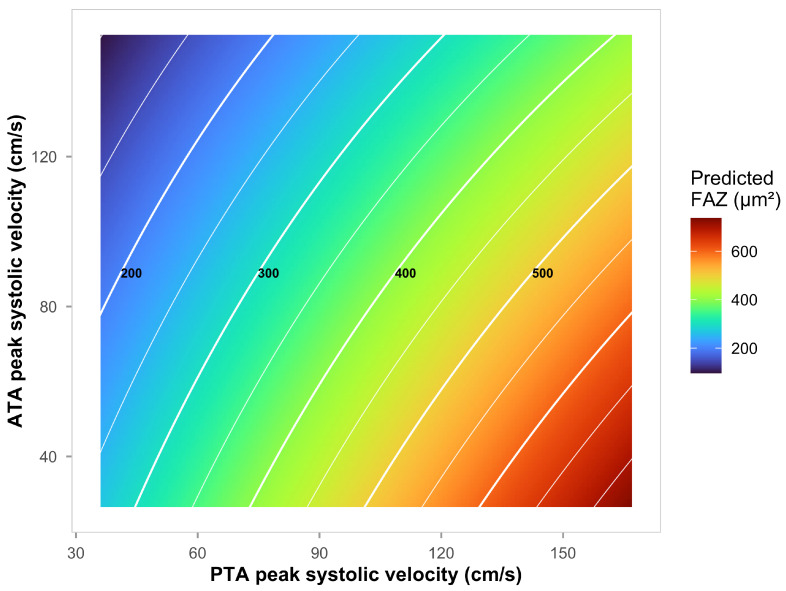
FAZ estimation using GAM interaction tensors (PTA:ATA). Surface shows estimated FAZ area (color scale: blue = small FAZ, red = large FAZ) as a function of posterior tibial artery PSV (*x*-axis) and anterior tibial artery PSV (*y*-axis). Contour lines connect regions of equal estimated FAZ. Higher posterior tibial velocity combined with lower anterior tibial velocity (top-left corner, yellow zone) corresponds to the largest FAZ areas, indicating worse retinal microvascular damage. Conversely, balanced velocities or lower posterior-to-anterior ratios (bottom-right, blue zone) correspond to smaller FAZ areas.

The unadjusted surface depicted in Figure 3 shows zones of higher estimated FAZ corresponding to unfavorable tibial velocity profiles (e.g., higher PTA with lower ATA). Isocontours every 50 µm^2^ aid clinical reading at common thresholds (300–400 µm^2^). Age is included as an adjuster in ordinary least squares (OLS), and age adjustment did not materially change the PT:AT association. Age amplified the PT:AT–FAZ association through stratified correlation analysis (FDR q = 0.024): patients aged <60 showed weak correlations (r = 0.23, *p* = 0.42), whereas those ≥60 showed strong ones (r = 0.55, *p* = 0.032). This age-dependent pattern suggests cumulative endothelial damage depletes vascular reserve capacity, making every additional hemodynamic insult more visible in retinal ischemia. The adjusted OLS effects in a multivariable model including age, diabetes duration, and HbA1c showed that PT:AT ratio remained independently associated with FAZ with an unstandardized coefficient of 93.42 µm^2^ per unit ratio (95% CI [18.37, 168.46], *p* = 0.017). Variance inflation was low (PT:AT VIF 1.03). Age adjustment did not materially alter the PT:AT association. An average PT:AT ratio of 1.28, a +0.5-unit increase in the ratio would correspond to an expected FAZ increase of roughly 47 µm^2^.

## 4. Discussion

### 4.1. Principal Findings

In our cross-sectional pilot study, we evaluated the relative strength of association between peripheral arterial markers and retinal microvascular damage. The posterior-to-anterior tibial peak systolic velocity ratio (PT:AT) demonstrated a moderate-to-strong association with foveal avascular zone (FAZ) area (r = 0.471, 95% confidence interval [CI] 0.13 to 0.71, *p* = 0.0086, false discovery rate q = 0.017), with complete concordance across five complementary analytical methods.

Our multi-method approach was chosen specifically to address limitations inherent in small-sample studies: Pearson correlation established baseline bivariate relationships; adjusted linear regression controlled for age, diabetes duration, and HbA1c as potential confounders; robust regression with bootstrap provided bias-corrected confidence intervals resistant to outlier influence; generalized additive models captured potential non-linear age-interaction patterns; and structural mediation analysis decomposed direct versus indirect pathways.

Convergent findings across all five methods strengthen causal inference despite the sample size. In contrast, the ankle-brachial index (ABI) showed a null association (r = −0.024, *p* = 0.901) with our hypothesis. PT:AT was significant (*p* = 0.0086) whereas ABI was non-significant (*p* = 0.901); this finding was consistent across all five analytical approaches.

A mediation analysis, using structural equation modeling, suggested that 93% of the estimated total association between PT:AT and FAZ was mediated through a direct peripheral-retinal pathway (average direct effect = 96.32 µm^2^, *p* = 0.047), with negligible transmission via the ophthalmic artery (average causal mediation effect = 6.79 µm^2^, *p* = 0.740; Figure 2). Exploratory mediation analysis indicated that age could operate predominantly directly on FAZ (68.9% of total effect). Serial mediation chains were minimal (<4%), supporting the hypothesis that age and tibial hemodynamics represent relatively independent mechanisms affecting FAZ through parallel pathways, consistent with systemic microvascular dysfunction affecting all vascular beds simultaneously.

We observed a significant age-dependent amplification of vascular coupling (false discovery rate q = 0.024). Patients aged < 60 years demonstrated weak correlations (r = 0.23, 95% CI −0.32 to 0.66), consistent with preserved arterial compliance and vascular reserve that may buffer hemodynamic pulsatility and protect the retinal and renal microvasculature. In contrast, patients aged ≥60 years exhibited substantially stronger correlations (r = 0.55, 95% CI 0.06 to 0.83), suggesting that age-related arterial stiffening transforms the vascular tree into a rigid conduit that transmits damaging pulsatile energy directly to fragile capillary beds, with reduced compensatory capacity to prevent microvascular injury. These findings indicate that tibial velocity profiling may be most clinically informative in older diabetic cohorts [25].

Mechanistically, age-related elastin fragmentation and collagen deposition reduce arterial cushioning function. This increases pulse pressure amplification from aorta to peripheral vessels—a process accelerated in diabetes by advanced glycation end-product cross-linking of arterial wall proteins.

A difference in PT:AT from the 10th to 90th percentile was associated with an estimated FAZ increase of 98.5 µm^2^, representing 25% of the 400 µm^2^ threshold defining clinically significant retinal ischemia [22,23]. This may suggest that PT:AT screening may offer greatest utility in patients aged ≥60 years, where diabetic vasculopathy and arterial aging intersect.

### 4.2. Comparison with Prior Literature

The magnitude of the observed PT:AT–FAZ correlation (r = 0.47) exceeds published associations between ABI and retinal vessel density (r = 0.23) in diabetic cohorts [26]. This supports our hypothesis that velocity-based functional hemodynamic metrics capture microvascular dysfunction with greater sensitivity than pressure-based structural indices, which are affected by vessel wall calcification. This finding aligns with established precedent for cross-organ vascular coupling. In the Atherosclerosis Risk in Communities cohort (N = 9371), retinopathy signs predicted incident peripheral arterial disease with adjusted hazard ratios of 2.0–3.0 for moderate disease and 3.0–6.0 for critical limb ischemia, with particularly robust associations in diabetes subgroups [9].

A systematic review of 28 studies confirms that peripheral arterial disease associates with structural and flow abnormalities on retinal imaging, highlighting the potential for optical coherence tomography angiography as a biomarker for peripheral vascular phenotyping [27]. Similarly, retinal microvascular abnormalities forecast cardiovascular events: narrower retinal arteriolar caliber independently predicts incident coronary heart disease (mean difference −5.55 µm, 95% CI −8.07 to −3.02) after adjusting for traditional cardiovascular risk factors [28]. Cross-organ coupling extends to renal microvascular beds. Meta-analysis with diabetes demonstrates that retinopathy strongly predicts diabetic kidney disease progression (pooled hazard ratio 2.42, 95% CI 1.70–3.45) [29]. Collectively, these prospective data support a systemic microvascular pathophysiology framework wherein accessible vascular measurements in one organ territory reflect pan-vascular endothelial health [3].

We found that PT:AT–FAZ association operates through a direct peripheral-retinal pathway rather than sequential hemodynamic transmission via the ophthalmic artery. Peripheral tibial velocity patterns and retinal capillary dropout may represent independent manifestations of a shared underlying microangiopathic state. To our knowledge, no prior studies have directly examined tibial artery velocity ratios in relation to FAZ area; our findings therefore represent hypothesis-generating evidence requiring independent replication before clinical application can be considered.

### 4.3. Mechanistic Interpretation

Three plausible biological mechanisms may explain the observed association. First, hyperglycemia-induced endothelial dysfunction operates simultaneously across all vascular beds through convergent molecular pathways. Chronic hyperglycemia triggers mitochondrial overproduction of reactive oxygen species, activating downstream damage cascades that impair nitric oxide bioavailability and disrupt vasomotor control [1,2]. Endothelial cells in both lower extremity arteries and retinal vessels express GLUT1 transporters that cannot downregulate uptake during hyperglycemia, rendering these beds equally vulnerable to metabolic toxicity [30,31]. This shared vulnerability provides a mechanistic basis for the observed parallel dysfunction.

Second, degradation of the endothelial glycocalyx may provide a unifying mechanistic link between peripheral hemodynamic alterations and retinal perfusion abnormalities. The glycocalyx functions as a critical mechanosensor and permeability barrier [32], and acute hyperglycemia induces systemic glycocalyx shedding, reflected by elevated circulating syndecan-1 and hyaluronan fragments that signify widespread endothelial injury [33,34]. Loss of this layer impairs shear stress transduction and flow distribution across vascular beds, potentially accounting for both altered tibial velocity profiles and retinal capillary dropout. Supporting this framework, arterial stiffness has been shown to correlate with foveal avascular zone enlargement via glycocalyx-mediated pathways [11]. Collectively, these findings suggest that the PT:AT ratio may serve as a macroscopic functional surrogate of systemic glycocalyx integrity, capturing concurrent increases in distal vascular resistance and retinal microvascular rarefaction.

Third, advanced glycation end-products (AGEs) accumulate systemically, binding to RAGE on endothelial cells and activating nuclear factor-κB inflammatory signaling [33]. This cascade promotes microvascular rarefaction through convergent pathways involving oxidative stress and impaired nitric oxide synthase activity [34,35]. The age-dependent amplification we observed (Figure 3; FDR q = 0.024) is consistent with cumulative AGE burden and progressive depletion of vascular reserve capacity documented in longitudinal diabetic cohorts [30]. Figure 3 displays the generalized additive model interaction surface, showing estimated FAZ area as a function of PT:AT ratio across the observed range. The surface demonstrates that FAZ enlargement increases progressively with higher PT:AT values, with the relationship more pronounced in older patients. The color gradient from blue (smaller FAZ) to red (larger FAZ) visualizes the clinical interpretation: patients with elevated PT:AT ratios exhibit greater retinal capillary dropout.

### 4.4. Superiority of Velocity-Based over Pressure-Based Metrics in Diabetes

The divergent associations for PT:AT (moderate-to-strong) versus ABI (null) underscore a fundamental methodological distinction relevant to diabetic populations. Medial arterial calcification—hydroxyapatite deposition in the arterial tunica media—stiffens tibial vessels and produces non-compressibility, yielding falsely elevated ABI values despite coexistent flow-limiting stenoses [12]. Medial arterial calcification prevalence reaches 57% in diabetes cohorts [35], and ABI sensitivity for peripheral arterial disease drops to approximately 35% in patients with diabetic neuropathy [36,37].

Contemporary guidelines explicitly recommend velocity-based alternatives when ABI exceeds 1.40 or when clinical suspicion persists despite normal pressure indices [13,14]. Doppler peak systolic velocity reflects actual hemodynamic perfusion through direct detection of red blood cell movement, unconfounded by vessel wall calcification [15,19]. The PT:AT ratio integrates multiple hemodynamic features to quantify distal microvascular resistance [17]. Abnormal PT:AT patterns may index microvascular rarefaction or endothelial dysfunction—processes invisible to pressure-based measurements.

Our multi-method validation showed consistent PT:AT superiority across five analytical approaches, with standardized effect estimates ranging from 0.5 to 0.7 for PT:AT versus near-zero for ABI (Figure 1). This convergent pattern supports the conceptual distinction between functional hemodynamic measures and structural pressure indices. This study employed methodological features that enhance the robustness of our preliminary findings.

First, we conducted comprehensive same-day vascular investigation across peripheral, ophthalmic, and retinal vascular beds, eliminating day-to-day physiological variability that could confound cross-organ associations. Standardized protocols included 15 min rest periods prior to Doppler assessment, consistent 60° angle correction, and three-cardiac-cycle averaging to minimize measurement error. The averaging of bilateral measurements reduces the influence of unilateral anatomical variants or focal disease, providing a more representative estimate of systemic hemodynamic status. Second, the five-method statistical analysis provides convergent validity that rejects method-specific artifacts or overfitting [21]. Third, the PT:AT ratio represents a composite hemodynamic signature integrating pulsatility and flow distribution patterns between two anatomically distinct tibial territories. Unlike single-vessel measurements that may reflect focal pathology, this ratio captures relative hemodynamic balance and may index systemic microvascular resistance changes more sensitively than isolated velocity or pressure parameters [17].

### 4.5. Limitations and Risk of Bias

The modest sample size (N = 30) provided adequate power for the primary hypothesis (80% power). Also, the cross-sectional design omits causal inference and temporal directionality. We cannot determine whether tibial hemodynamic alterations precede, follow, or develop concurrently with retinal microvascular damage. Although biological plausibility supports shared systemic pathophysiology, the observed associations may reflect unmeasured confounding or bidirectional relationships. Longitudinal studies are essential to establish predictive validity and temporal sequencing. Although we used standardized protocols and an experienced operator, Doppler measurements and FAZ quantification are susceptible to segmentation and motion artifacts [23,38]. Residual confounding from unmeasured variables cannot be excluded. Despite adjustment for age, duration of diabetes, and glycated hemoglobin, unmeasured factors, such as specific classes of medications, smoking pack-years, physical activity, and genetic susceptibility may still influence the results. The interaction between disease duration and the PT:AT–FAZ association could not be formally tested due to retrospective uncertainty in diabetes onset timing and insufficient statistical power for stratified analyses. Furthermore, the selection bias in our cohort may limit generalizability; the single-center recruitment from a specialized diabetic foot clinic may enrich for patients with more severe peripheral disease or greater health engagement than community-based populations. Lastly, a limitation of our PT:AT metric is the exclusion of the peroneal artery, which is often the last patent vessel in diabetic PAD (‘peroneal sparing’). Future studies should evaluate multi-vessel indices that incorporate peroneal flow to provide a more comprehensive assessment of angiosome-specific perfusion.

### 4.6. Clinical Implications and Future Research Directions

If these preliminary findings are validated in larger prospective cohorts, integrating PT:AT assessment into existing diabetic foot screening protocols could facilitate simultaneous retinopathy risk stratification. Current guidelines recommend annual foot examination [17] calculating PT:AT requires minimal additional time and uses existing equipment. This approach may offer utility in resource-limited settings where ophthalmology capacity is restricted, enabling prioritized retinal screening for high-risk patients.

## 5. Conclusions

In our data, the velocity-based tibial artery hemodynamics (PT:AT ratio) demonstrated a moderate-to-strong association with retinal microvascular damage (r = 0.47, 95% CI [0.13, 0.71], *p* = 0.009), while the mediation analysis suggesting this association is independent from the ophthalmic artery resistivity index. In contrast, the pressure-based ABI showed no association. Our results provide preliminary evidence for PT:AT as a potential biomarker of systemic microvascular dysfunction. Prospective validation in larger, more diverse cohorts is necessary.

## Figures and Tables

**Table 1:** Patient characteristics.

Variable	N = 30 (%); Mean (SD); Median [Q1, Q3]
Sex	
Male	17 (59%)
Female	12 (41%)
Age (years)	61.0 (9.3); 59.5 [55.0, 68.0]
BMI (kg/m^2^)	29.9 (4.4); 29.0 [28.0, 33.0]
Diabetes duration (years)	13.2 (8.2); 10.0 [6.0, 17.0]
Hypertension	25 (83%)
Current/former smoker	16 (53%)
Nephropathy	58.8 (9.4); 59.0 [55.0, 66.0]
Cardiac disease	34.7 (7.1); 35.5 [28.0, 40.0]
Neuropathy stage	
None	18 (69%)
Moderate	4 (15%)
Severe	4 (15%)
Insulin therapy	20 (67%)
Oral antidiabetics	28 (93%)
Foot ulcer history	12 (40%)
Amputation history	4 (13%)
HbA1c (%)	7.2 (1.0); 7.1 [6.5, 7.4]
Fasting glucose (mg/dL)	161.7 (42.6); 159.5 [122.0, 188.0]
LDL cholesterol (mg/dL)	124.8 (34.8); 123.0 [104.0, 144.4]
HDL cholesterol (mg/dL)	46.0 (14.4); 43.0 [36.0, 54.0]
Triglycerides (mg/dL)	147.3 (48.6); 148.0 [123.0, 168.0]
eGFR (mL/min/1.73m^2^)	87.1 (15.6); 89.0 [73.0, 97.1]
Creatinine (mg/dL)	0.9 (0.2); 0.9 [0.7, 1.0]
PT:AT ratio	01.28 (0.62); 1.11 [0.99, 1.42]
Ankle-brachial index (worst)	0.97 (0.19); 1.03 [0.82, 1.11]
Ophthalmic artery RI (max)	0.78 (0.05); 0.79 [0.75, 0.83]
FAZ area (µm^2^, worst eye)	343.3 (134.9); 343.7 [236.4, 408.4]

Note. Values are mean (SD); median [Q25, Q75] for continuous variables and N (%) for categorical variables. Abbreviations: PT:AT = posterior-to-anterior tibial PSV ratio; ABI = ankle-brachial index; RI = resistance index; FAZ = foveal avascular zone; eGFR = estimated glomerular filtration rate; Q25 = 25th percentile; Q75 = 75th percentile.

**Table 2:** Clinical interpretation guide—predicted FAZ area by PT:AT ratio quartiles.

PT:AT Category	PT:AT Value	Predicted FAZ (µm^2^)	Clinical Significance
Low (Q1)	0.99	313	Below median retinal damage
Median	1.11	326	Typical diabetic cohort
High (Q3)	1.41	357	Elevated risk (δ43 µm^2^ vs. Q1)
Very High (P90)	1.81	398	High risk (δ98 µm^2^ vs. P10)

Note. Estimated FAZ values calculated from unstandardized linear regression (slope = 103.8 µm^2^ per unit PT:AT increase). Q1/Q3 = first/third quartile; P10/P90 = 10th/90th percentile. The 400 µm^2^ threshold indicates clinically significant retinal ischemia associated with retinopathy progression.

## Data Availability

Deidentified data and analysis codes are available upon reasonable request. The discovery of the coefficient error emphasizes the importance of data transparency.

## Abbreviations

The following abbreviations are used in this manuscript:

DR	Diabetic retinopathy
PAD	Peripheral arterial disease
OCT-A	Optical coherence tomography angiography
FAZ	Foveal avascular zone
ABI	Ankle-brachial index
MAC	Medial arterial calcification
PSV	Peak systolic velocity
PT:AT	Posterior-to-anterior tibial peak systolic velocity ratio
EDV	End-diastolic velocity
RI	Resistance index
OA	Ophthalmic artery
VIF	Variance inflation factor
GAM	Generalized additive model
OLS	Ordinary least squares
ADE	Average direct effect
ACME	Average causal mediation effect
AGEs	Advanced glycation end-products
RAGE	Receptor for advanced glycation end-products
